# Correlates of Oral Health-Related Quality of Life Among Chinese American Family Dementia Caregivers

**DOI:** 10.1093/geroni/igaf122.1024

**Published:** 2025-12-31

**Authors:** Weiyu Mao, Bei Wu, Yaolin Pei

**Affiliations:** University of Nevada, Reno, Reno, Nevada, United States; NYU Shanghai, Shanghai, China (People’s Republic); The University of Texas at Austin, Austin, Texas, United States

## Abstract

Oral health-related quality of life (OHRQoL) is a multidimensional concept that reflects how oral health influences one’s overall well-being and daily functioning. There is scarce empirical evidence on the correlates of OHRQoL in dementia caregivers. Guided by the Andersen’s model, this study investigated correlates of OHRQoL among family dementia caregivers in Chinese American communities. Data were from a pilot study on Chinese American family dementia caregivers in New York City between November 2021 and June 2022. Purposive sampling was used to recruit caregivers to participate in an online or telephone survey. Current caregivers (n = 76) were included. The dependent variable was caregiver oral health-related quality of life, measured by three items with a 5-point response set: 0 = never, 1 = hardly ever, 2 = occasionally, 3 = fairly often, and 4 = very often (Cronbach’s alpha = 0.71). Summated scores were used, and higher scores suggest worse OHRQoL. Results from bivariate analyses suggest that caregivers having financial difficulty tended to report worse OHRQoL. Those caring for someone with moderate dementia tended to report better OHRQoL. Caregivers with longer length of stay and those with more natural teeth tended to report better OHRQoL. Results from multiple regressions suggest that having financial difficulty was significantly associated with worse OHRQoL in caregivers. Having more natural teeth was significantly associated with better OHRQoL in caregivers. This study highlights the importance of studying OHRQoL in dementia caregivers and offers empirical evidence to support intervention strategies to improve oral health in this vulnerable population.

